# Generation and Characterization of an scFv Directed against Site II of Rabies Glycoprotein

**DOI:** 10.4061/2011/652147

**Published:** 2011-10-05

**Authors:** Shukra M. Aavula, Sridevi V. Nimmagadda, Neelakantam Biradhar, Samuel Sula, Dev Chandran, Rajendra Lingala, Srinivasan Alwar Villuppanoor

**Affiliations:** Research and Development Center, Indian Immunologicals Limited, Rakshapuram, Gachibowli, Hyderabad 500032, India

## Abstract

Recombinant antibody phage display technology is a vital tool that facilitates identification of specific binding molecules to a target enabling the rapid generation and selection of high affinity, fully human, or mouse antibody product candidates essentially directed towards disease target appropriate for antibody therapy. In this study, a recombinant single-chain Fv antibody fragment (scFv) A11 was isolated from immune spleen cells obtained from mice immunized with inactivated rabies virus (Pasteur strain) using standard methodology and was characterized for its specificity towards the rabies virus glycoprotein. Epitope mapping using peptide libraries and truncated glycoprotein polypeptides suggested that A11 bound to the antigenic site II of rabies glycoprotein against which a majority of rabies virus neutralizing antibodies are directed. The use of the above technology could, therefore, allow development of scFvs with different specificities against the rabies glycoprotein as an alternative to the more cumbersome protocols used for the development of monoclonal antibodies.

## 1. Introduction

Rabies is a viral, zoonotic, and invariably fatal neuroinvasive disease of humans caused by the bite of rabid animal. More than 55,000 deaths occur annually worldwide in spite of the use of postexposure therapy preventive measures, making rabies one of the major causes of human mortality despite significant scientific progress [1, 2]. Administration of antivirus immunoglobulins includes the use of both human and equine antirabies immunoglobulins along with vaccination and is the only strategy recommended by WHO for the postexposure prophylactic treatment of rabies [3]. Most of these immunoglobulins are plasma-derived, polyclonal products obtained from human and animal donors vaccinated against rabies which can be produced in limited amounts and suffer from potential drawbacks such as batch-to-batch variation and the risk of contamination from other pathogens [4, 5]. 

Several conventional monoclonal antibodies (MAbs) have been generated against different viruses [6] and their utilization tends to be limited in clinical applications because of possible viral contamination and high cost involved in MAb preparations. To overcome these problems, generation of single chain antibody fragments (scFv) through phage display technology has been utilized as the methodology of choice [7–9]. Use of phage display technology as a powerful *in vitro* tool for production of therapeutically important antibodies against viral pathogens on the surface of bacteriophage provides an efficient method for isolation and screening of a diverse set of human and nonhuman antibodies from immunized or naïve volunteers against various infectious diseases. These antibodies have the potential of being used as immunoprophylactic/therapeutics against different disease-causing agents [10–15]. 

In the present study, an immune scFv library was constructed using RNA isolated from splenocytes of mice immunized with an inactivated rabies vaccine. Specific mouse scFv fragment was affinity selected from this library using inactivated rabies virus. The selected scFv A11 was shown to recognize an epitope on rabies glycoprotein (GP) antigenic site II using immune phage display library, which was further confirmed through its reactivity to truncated polypeptides of PV GP containing the site.

## 2. Material and Methods

### 2.1. Antigens, Antibodies, and Animals

Inactivated Pasteur virus rabies vaccine (AbhayRab) and purified rabies virus were obtained from Human Biologicals Institute, Ooty, India. Chikungunya antigen and its polyclonal antibodies, hepatitis A antigen and its polyclonal antibodies were procured from virology laboratory, Indian Immunologicals limited (IIL). Hepatitis B surface antigen and its monoclonal antibody (1F6) were obtained from hybridoma laboratory, IIL. Splenocytes were obtained from BALB/c mice immunized with Abhayrab (Pasteur strain); purified native glycoproteins from Pasteur virus (PV GP) were obtained from the hybridoma Laboratory, IIL, for construction of immune antibody phage display library.

### 2.2. Bacterial Strains, Vectors, and Chemicals

The bacterial strains used for protein overexpression in *E. coli *BL21(DE3), M13K07 helper phage for recombinant phage production, and all molecular biology reagents were purchased from invitrogen (Carlsbad, USA). The phagemid vector pCANTAB 5E used for cloning and expression of the scFvs on phage coat protein (pIII) was kindly provided by Dr. Sandra Saptas (CSIRO, Australia). The bacterial expression vector pET 20b was purchased from Novagen (Madison, USA). The plasmid mini-prep kit for isolation of plasmid, gel extraction kit for extraction of DNA, PCR purification kit, HiFi Taq DNA polymerase, PCR reagents, and Ni-NTA agarose used for the purification of His- tagged proteins were purchased from Qiagen (Hilden, Germany). The bacterial strain (*E. coli* TG1) used for the propagation of phagemid vector pCANTAB 5E was purchased from Stratagene, USA. Nuclease-free water and T4 DNA Ligase were purchased from GeNei, India. Glutathione agarose used for purification of GST tagged proteins and all other fine chemicals used were purchased from Sigma (USA).

### 2.3. Isolation of Total RNA and cDNA Synthesis

Total RNA was isolated from about 10 × 10^6^ spleen cells collected from immunized mice using TRIzol reagent (Invitrogen) and resuspended in nuclease-free water. RNA was quantified by Biophotometer (Eppendorf, USA) and was subjected to cDNA synthesis using random hexamers and Thermoscript reverse transcriptase (RT)-PCR kit (Invitrogen) according to the manufacturer's instructions. The cDNA was stored at −20°C until further use.

### 2.4. PCR Amplification of Heavy and Light Chain Variable Genes

PCR reaction was set up in a total volume of 50 *μ*L containing 5 *μ*L of cDNA, 5 *μ*L of 10x HiFi Taq DNA polymerase reaction buffer, 1 *μ*L of 10 mM dNTP's, 1 *μ*L (10 pmol/*μ*L) of each forward and reverse universal, degenerate primer (synthesized by MWG, India) (Table 1) [16], and 1.25 units of HiFi Taq DNA polymerase enzyme. After chilling the reaction mix on ice, it was transferred to a master cycler (Eppendorf) and cycled 7 times for 60 sec at 92°C, 30 sec at 63°C, 50 sec at 58°C, and 60 sec at 68°C followed by 60 sec at 92°C, 60 sec at 63°C, and 60 sec at 68°C for 23 cycles. Finally the extension cycle was increased to 10 min at 68°C. Amplifications were repeated several times, and the final variable light chain (VL) and variable heavy chain (VH) products were pooled and gel purified.

### 2.5. Assembly of VH and VL and Cloning into Phagemid Vector

Variable domains were amplified as described above, gel purified, and assembled by a linker (Gly_4_Ser)_3_ using splicing by overlap extension polymerase chain reaction (SOE PCR) with VL forward and VH reverse primers (Table 1). Purified VH and VL were used in PCR for 14 cycles in the absence of primers. The amplified PCR product was reused as the template for amplifying (94°C for 60 sec, 63°C for 60 sec, 68°C for 2 min, 34 cycles) scFv which yielded a product of size ~750 bp using forward light chain primer (with *Nco*I site) and reverse heavy chain primer (with *Not*I site) for 34 cycles. The PCR product containing the amplified scFv was gel purified by gel extraction kit and cloned into pCANTAB 5E vector, which was double, digested with *Nco*I and *Not*I restriction enzymes according to the manufacturer's instructions (New England Biolabs, MA, USA). The digested products were further purified using a PCR purification kit, and ~98 ng of the digested scFv fragment was ligated into ~100 ng of digested phagemid vector (pCANTAB 5E) in a total volume of 20 *μ*L using T4 DNA ligase. The ligated product was electroporated into electrocompetant *E. coli* TG1 cells (Stratagene, USA, transformation efficiency of 1 × 10^9^/*μ*g of DNA) using an electroporator (Gene pulser Xcell, Bio-Rad, USA), and the transformed bacteria were plated on 2xYT agar.

### 2.6. Preparation of Phage Library

The resultant lawn of bacterial cells that was obtained after transformation on selective antibiotic media were scraped off the agar plates into 15 mL 2xYT media (2x Yeast extract, Tryptone) and stored at −70°C in aliquots of 1 mL containing 20% glycerol. An aliquot was thawed and diluted with 9 mL of 2xYT containing 50 *μ*g/mL of ampicillin and 2% glucose (2xYT-AG). These bacterial cells were infected with 6 × 10^10^ plaque forming units (pfu) of M13K07 (Invitrogen) and the infected bacteria were incubated in an orbital shaker at 37°C for 2 h. The cells were collected by centrifugation at 5000 rpm for 10 min and resuspended in 10 mL of 2xYT containing 100 *μ*g/mL of ampicillin and 50 *μ*g/mL of kanamycin. The culture were grown overnight at 37°C before harvesting. The supernatant-containing phages were filtered through a 0.45 *μ*m filter and concentrated by precipitation with 1/5th volume polyethylene glycol solution (20% PEG 6000, 2.5 M NaCl) for 1 h on ice. The precipitated phagemids were centrifuged (20 min, 6000 × g, 4°C) and resuspended in phosphate-buffered saline (PBS). Following another brief centrifugation at 4°C for 5 min at 13000 × g, the cellular debris was removed. The concentration of the infectious phage particles was determined by infecting log phase *E. coli *strain TG1 cells with serially diluted phages ranging from 10^2^ to 10^8^, incubated in an orbital shaker at 37°C for 10 min and plated on 2xYT-AG agar plates. The phage stocks were stored at 4°C until use.

### 2.7. Biopanning of Phage Display Library against PV GP

Maxisorp Immunotube was (Nunc, Denmark) coated overnight at 4°C with PV GP diluted in 50 mM of sodium carbonate-bicarbonate buffer, pH 9.6. After washing the tube 4 times with phosphate-buffered saline containing 0.05% Tween-20 (PBS-T), blocking was done with 1% bovine gelatin in PBS-T (1% BGPBS-T) and incubated at 37°C for 2 h. The tube was washed as mentioned above and incubated with titrated phages at 37°C for 2 h. Finally the tube was washed with PBS-T 10 times followed by 10 washes with PBS. Log phase *E. coli *TG1 cells were added to the tubes and incubated at 37°C to allow infection by bound phages. Infected culture was twofold serially diluted and plated on 2xYT-AG agar plates to determine the titer of phage. The remaining culture was plated and incubated at 37°C for overnight. The cells were scraped and infected with helper phage M13K07 and subjected to an additional rounds of panning. Further panning was done for selection of high affinity phages with higher stringency using higher concentrations of Tween-20 that ranged from 0.05% to 0.1% and also the number of washes from 10 to 20 times.

### 2.8. Screening Specific Binding Clones by Phage ELISA

ELISA was performed to check the binding ability of phage to rabies virus as described by Clackson et al. [17]. Individual colonies after third round of biopanning were inoculated into 100 *μ*L of 2xYT containing 100 *μ*g/mL ampicillin and 2% glucose (2xYT-AG) and incubated in an orbital shaker at 37°C for overnight. 10 *μ*L was subcultured to 100 *μ*L/well of 2xYT-AG media and incubated in an orbital shaker at 37°C till they reached an O.D_600_ of 0.6. M13K07 helper phage at a concentration of 2.5 × 10^10^ pfu/mL was added and the cultures were further incubated in an orbital shaker for 2 h and then centrifuged at 4000 rpm for 20 min. The bacterial pellet was resuspended in 2xYT containing 100 *μ*g/mL ampicillin, 50 *μ*g/mL kanamycin, and 2% glucose and grown at 30°C overnight in an orbital shaker. Cells were pelleted, and the supernatant-containing phages were collected for analysis in phage ELISA. Briefly, microtiter plates (Nunc, Roskilde, Denmark) were coated with 100 *μ*L/well of 0.05 M carbonate-bicarbonate buffer (pH 9.6) containing 5 *μ*g/mL of rabies purified antigen and incubated overnight at 4°C. Blocking was performed using 2% bovine gelatin for 1 h at 37°C using PBS-T followed by incubation with phages at a concentration of 10^11^/100 *μ*L of bacterial supernatant/well for 2 h at 37°C. Background noise was estimated using nonspecific phages in an uncoated microtiter plate. Binding of the phage particles to antigen was detected with the horseradish peroxidase-(HRP-)conjugated anti-M13 mouse antibody (Pharmacia Biotech, dilution 1 : 5000) and followed by 3, 3′, 5, 5′ tetramethylbenzidine (TMB). The plate was incubated at 37°C for 10 min, and the reaction was stopped by addition of 1.25 M H_2_SO_4_. The absorbance was measured at 450 nm using a microplate reader (BIO-TEK, USA).

### 2.9. Restriction Mapping and Sequence Analysis

The diversity of the selected recombinant clones was checked by amplifying the scFv insert using primers light forward (LB) and heavy chain reverse (HF) followed by digestion with restriction enzyme *Bst*NI. The banding pattern of the restriction digestion was analyzed by 3% agarose gel electrophoresis. Phagemid DNA was isolated using Qiagen miniprep kit. Each scFv construct was sequenced using S1 and S6 sequencing primers (Amersham Biosciences, USA). Nucleotide sequence was determined using the standard protocols from Big Dye Terminator v3.1 cycle sequencing kit (Applied Biosystems, USA) in conjunction with AB3130XL automated genetic analyzer (Applied Biosystems, USA). The sequences were analyzed by using IMGT/ V-QUEST software [18].

### 2.10. Expression and Purification of Soluble Antibody Fragments

Phages, which showed reactivity with rabies virus were used to isolate plasmid DNA with a commercial kit. The scFv was amplified with primers containing *Eco*RI and *Not*I sites at 5′ and 3′ ends, gel purified, and cloned into bacterial expression vector pET 20b and transformed into *E. coli* BL21 (DE3) cells. ScFv production was induced by addition of 1 mM isopropyl-*β*-D-thiogalactopyranoside (IPTG) and incubated for 4 h at 30°C. The bacterial pellet was collected by centrifugation at 5000 × g for 20 min at 4°C, resuspended in lysis buffer and sonicated. The supernatant was purified by immobilized metal affinity chromatography (IMAC). An IMAC column (5 mL) was equilibrated with 10 column volumes of 50 mM Tris-HCl, 155 mM NaCl, pH 7.6 (equilibration buffer). The supernatant was loaded to the column at a flow rate of 1 mL/min and washed with 20 column volumes of washing buffer containing equilibration buffer with 30 mM imidazole, pH 7.6. Bound scFv was eluted with 5 column volumes of elution buffer containing equilibration buffer with 300 mM imidazole, pH 7.6, as fractions of 1 mL each. Purified protein was dialysed extensively against PBS. Protein concentration was determined by the BCA assay kit (Sigma, USA) and stored at −20°C till further use.

### 2.11. Electrophoresis and Immunoblot Analysis

The purified scFv was electrophoresed on a 12% SDS-PAGE [19]. The separated proteins were transferred to a PVDF membrane (Hybond-C, Amersham Biosciences, USA) using transblot apparatus (Bio-Rad, USA) following manufacturer's instructions. The blot was probed with His-probe (Pierce, USA) and developed using 0.05% of DAB (Sigma, USA) and 0.03% hydrogen peroxide in PBS.

### 2.12. Determination of Specificity of the scFv A11 against PV GP

The specificity was determined by (a) immuno-blot transfer assay, (b) Sandwich ELISA to determine the reactivity of A11 towards PV GP, (c) Competitive ELISA to assess the competition of A11 with monoclonal M5B4, and (d) Binding specificity of A11 to unrelated viruses. All the procedures were done as described by Sridevi et al. [20].

### 2.13. Epitope Mapping of A11 Fragment

The A11 antibody-bound epitope was mapped with a constrained phage display peptide library of random peptide 7 mers fused to a minor coat protein (pIII) of M13 phage. Maxisorp Immunotube (Nunc, Denmark) was coated overnight at 4°C with purified A11 (50 ng/well) diluted in 0.1 M NaHCO_3_ (pH 8.6). The tubes were washed with PBS-T 5 times and incubated for 2 h at 4°C with blocking buffer, 1% BGPBS-T. The tube was washed as mentioned above and incubated with 4 × 10^10^ of constrained Ph.D.-C7C peptide phage library (New England Biolabs) at room temperature for 2 h. The tube was washed and bound phages were eluted by disrupting the binding interactions with 0.2 M glycine (pH 2.2), 1 mg/mL BSA, and neutralized with 1 M Tris-HCl (pH 9.1). The bound phages were subjected to three rounds of biopanning as mentioned above, and the eluted phages from third round were titrated by infecting *E. coli* strain ER2738. The clones obtained after the final round of panning/titrations were sequenced, and analysis of aminoacid sequence of the presented peptide was deduced to determine the consensus-binding motif.

### 2.14. Amplification of Glycoprotein Gene and Cloning into pGEX 4T1

To determine the binding site of A11 in PV GP, the glycoprotein gene was truncated and amplified as five different gene products, namely, E1, E2, E3, E4, and E5, (Table 2(a)) using gene specific primers and PCR (Table 2(b)). E1–E5 and pGEX 4T_1_ were digested using the restriction enzymes *Bam*HI and *Eco*RI according to the manufacturer's instructions (New England Biolabs, MA, USA) and purified using a gel purification kit. ~45 ng of each of the digested epitope fragments E1–E5 were ligated into ~100 ng of digested pGEX 4T_1_ vector in a total volume of 20 *μ*L using T4 DNA ligase, and the ligated products were transformed into chemically competent *E. coli* strain of TOP 10 cells. The plates were incubated overnight at 37°C and individual colonies were picked from each plate, inoculated into the medium containing ampicillin, and grown at 37°C in an orbital shaker for overnight. The overnight grown culture was used for isolation of plasmids using plasmid mini-prep kit from QIAGEN, and positive clones were confirmed by restriction double digestion and sequence analysis.

### 2.15. Expression and Purification of Glycoprotein Epitope Genes

pGEX 4T_1_ E1–E5 plasmids were transformed into BL21 (DE3) cells of *E. coli* strain. Individual colonies were picked, and each of the culture expressing epitopes E1–E5 regions of glycoprotein was induced by addition of 1 mM IPTG and incubated for 4 h at 30°C. The bacterial pellet was collected by centrifugation at 5000 × g for 20 min at 4°C and resuspended in lysis buffer and sonicated. The supernatant was purified by glutathione agarose column. Purified protein was dialysed extensively against PBS. Protein concentration was determined by the BCA assay kit (Sigma, USA) and stored at −20°C for further characterization.

### 2.16. Indirect ELISA for Epitope Specificity

A microtiter plate (Nunc, Denmark) was coated with 100 ng/well of E1–E5 in 50 mM carbonate-bicarbonate buffer (pH 9.6), respectively, and incubated overnight at 4°C. The plate was washed thrice with PBS-T and blocked with 1% BGPBS-T followed by washing with PBS-T. 20 ng/100 *μ*L of A11 was added in the first well, serial diluted, and incubated at 37°C for 1 h. The plate was washed five times with PBS-T and dried. The binding of the A11 with different epitopes of PV GP was detected by addition of His-probe and a chromogenic substrate TMB. The reaction was stopped by addition of 1.25 M H_2_SO_4_, and the absorbance was measured at 450 nm using a microplate reader (BIO-TEK, USA). The experiment was done in triplicates to evaluate the concentration-dependent binding activity of A11 towards different epitopes of PV GP.

## 3. Results

### 3.1. Amplification and Cloning of VH and VL Genes to Form scFv Phage Display Cassette

**Figure 1:**
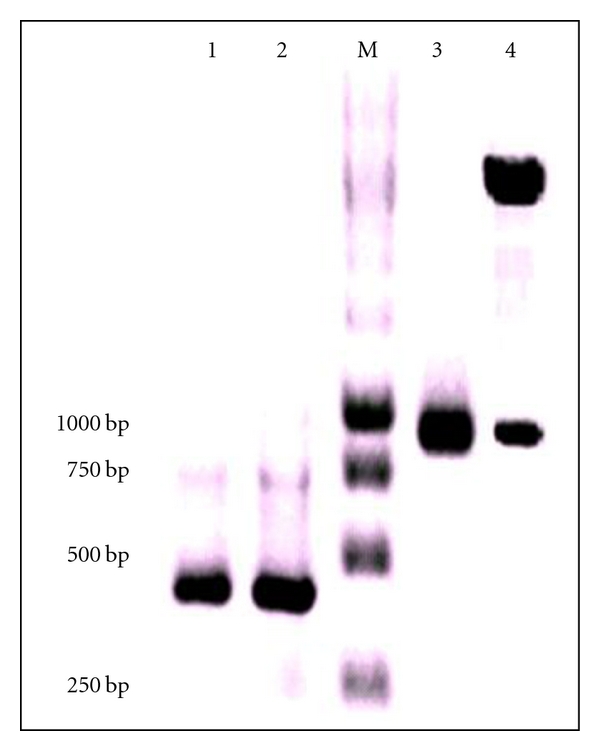
Agarose gel electrophoresis analysis of PCR-amplified products from immunized splenocytes. Lane M shows the DNA ladder and Lanes 1, 2, and 3 show the variable heavy, variable light chain genes, and assembled PCR products, respectively. Lane 4 is the recombinant expression cassette after *Nco*I and *Not*I digestion showing release of scFv product.

Total RNA was isolated from immunized mice spleen cells and cDNA was synthesized. The cDNA was used as a template for the PCR amplification of the VH and VL genes by using universal primers (Table 1), which yielded a 321 bp and 303 bp amplicon as shown in Figure 1, Lanes 1 and 2, respectively. The resultant VH and VL were assembled into a single chain fragment using a peptide linker (Gly_4_Ser)_3_ using SOE PCR which could be visualized as a 750 bp amplicon on the agarose gel as shown in Figure 1, Lane 3. The scFv was digested by *Nco*I/*Not*I and ligated into the phage display vector pCANTAB 5E to form the cassette yielding a library of 4.2 × 10^7^ pfu/mL. The presence of scFv in the library was analyzed by *Nco*I/*Not*I digestion as shown in Figure 1, Lane 4.

### 3.2. Selection of Phage Displaying Antirabies Recombinant Antibodies by Biopanning

**Figure 2:**
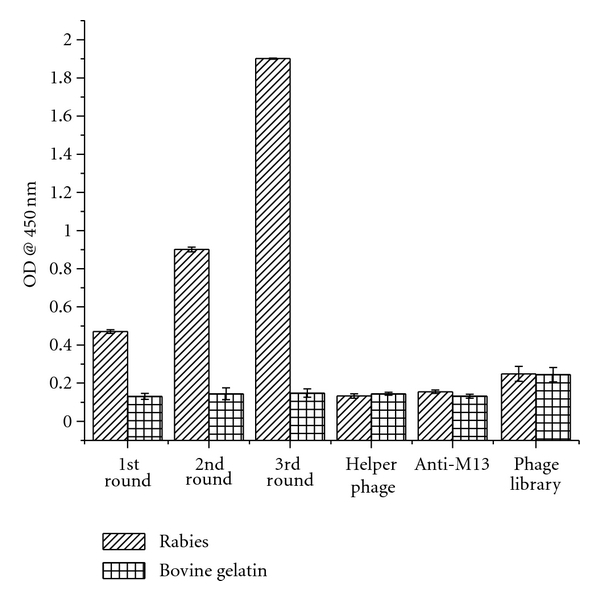
Phage ELISA showing the enrichment of phages specific for rabies virus glycoprotein during the panning cycles. After each round of panning, the output phages were added to microtiter wells with 200 ng of PV GP or bovine gelatin. Anti-M13 and helper phage was used as a control to check the back ground value. Bound phages were detected by horseradish peroxidase (HRP) conjugated anti-M13 antibody.

An scFv phage display library containing 1 × 10^12^ pfu/mL of independent clones was subjected to biopanning for selection of scFvs against PV GP. Enrichment of antigen-specific binding phage was measured by performing the three rounds of panning, which resulted in 1 × 10^3^, 1 × 10^5^, and 1 × 10^9^ pfu of eluted phage in first, second and third rounds of panning, respectively. Antigen-binding activity of the pooled phages from each round of the panning was analyzed by phage ELISA, resulting in the successful enrichment of rabies-virus-specific binders as shown in Figure 2. Eluted phages from round 3 were used to infect *E. coli* TG1 to produce phage antibodies, and the supernatants containing phage antibodies from 96 individual clones were tested by phage ELISA against PV GP, to select antigen-specific phage scFvs. Of the 96 clones tested, 30 showed positive binding to rabies virus. PCR analysis confirmed that the selected clones contained an insert corresponding to the size of an scFv fragment. DNA sequence analysis of two strongest binding clones, A11 and D11, showed similar VH and VL sequences. All further studies were done using A11.

### 3.3. DNA Fingerprint Analysis Using Restriction Fragment Length Polymorphism (RFLP)

To determine the diversity of the library, PCR-amplified scFv fragments from 30 individual clones of scFv library were analyzed by *Bst*NI fingerprinting. Each scFv showed a different digestion pattern indicating variations in *Bst*NI recognition sites confirming diversity amongst randomly selected clones. To confirm that the scFvs consisted of VH and VL chains of immunoglobulin molecules and to further analyze library diversity, DNA sequence analysis was performed on 30 randomly selected clones obtained from an scFv library. All scFv sequences were analyzed using the web-based NCBI Ig BLAST program (http://www.ncbi.nlm.nih.gov/igblast/) and IMGT/V-QUEST software to approximate framework and complementarity determining regions. In the library sequenced, the majority of VH and VL chains had open reading frames encoding full-length VH and VL chains (data not shown).

### 3.4. Expression and Purification of Soluble scFv A11

**Figure 3:**
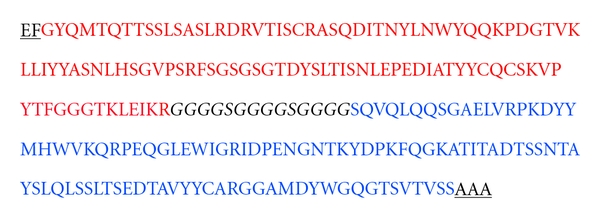
Amino acid sequence of anti-rabies mouse scFv containing VL, linker peptide and VH. The linker peptide is marked in italics. The restriction enzyme sites for cloning of the scFv gene are underlined.

**Figure 4:**
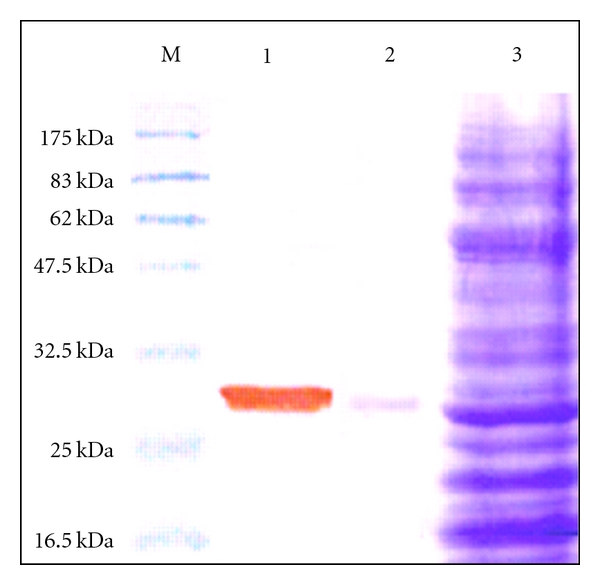
Detection of recombinant A11 on 12% sodium dodecyl sulphate-polyacrylamide gel electrophoresis. Lane M shows protein molecular size standard. Lane 1 is a purified protein transferred onto PVDF membrane and probed with His-probe and developed using DAB substrate. Lanes 2 and 3 are a purified protein and crude lysate stained with coomassie brilliant blue R-250.

Sequence analysis of scFv A11 by International ImmunoGeneTics information system (IMGT) revealed the presence of 223 amino acids as shown in Figure 3. ScFv A11 was transformed into *E. coli* BL21 (DE3) for production of soluble scFv for further characterization. The purified protein eluted fraction of scFv shows a ~28 kDa band on both SDS-PAGE and immunoblot analysis as indicated in Figure 4.

### 3.5. Reactivity of A11 with Rabies Virus Glycoprotein

**Figure 5:**
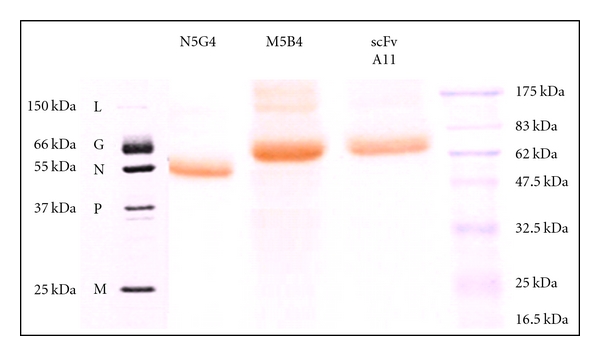
Reactivity of the A11 with the rabies virus glycoprotein in immuno-transfer blot analysis. Lane N5G4 was developed using antibody specific to nucleoprotein of rabies virus. Lane M5B4 was developed using antibody specific to glycoprotein site III of rabies virus. Lane A11 was developed using A11 specific to glycoprotein of rabies virus. Lane M shows protein molecular size standard.

Immunoblotting of A11 and M5B4 against the rabies virus structural proteins resolved in a 10% gel by nonreducing SDS-PAGE clearly indicated binding to a ~66 kDa protein corresponding to PV GP as indicated in Figure 5. The PV-NP-specific MAb-N5G4 was used as a negative control and it bound to a ~55 kDa protein corresponding to rabies virus nucleoprotein (PV NP).

### 3.6. Sandwich ELISA

**Figure 6:**
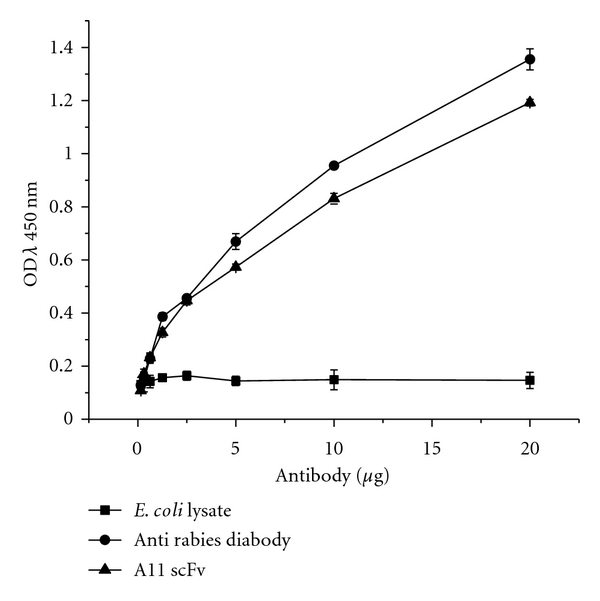
Analysis of antigen binding affinity of A11 by ELISA to evaluate the activity of A11 to rabies virus glycoprotein. Negative control used for ELISA is *E. coli* lysate, and positive control is antirabies diabody.

Sandwich ELISA was performed to determine the reactivity of the A11. Titration of the PV GP against a constant dilution of A11 revealed a concentration-dependent reduction of the binding signal as shown in Figure 6. The control showed no reactivity in ELISA indicating the specificity of the A11.

### 3.7. Competitive ELISA to Determine Site Specificity of A11

**Figure 7:**
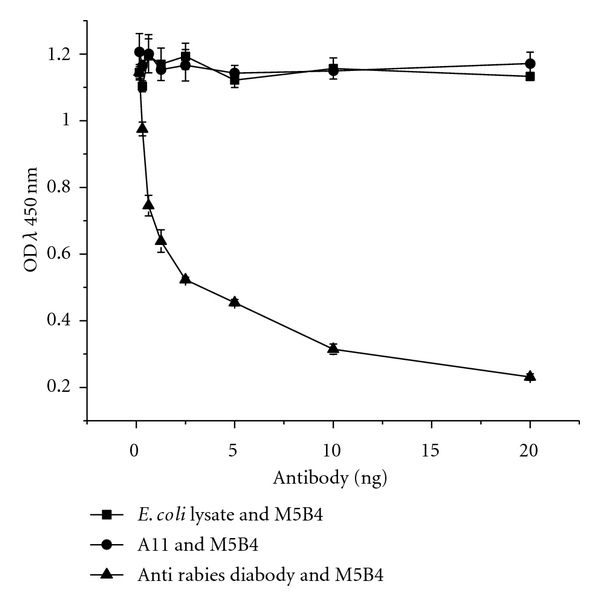
Competitive ELISA using the A11 and the RV GP-specific human antirabies diabody and mouse Mab M5B4 specific for site III.

Competitive ELISA was performed to determine the competition between M5B4 [21] and the A11 for the same site on PV GP. No competition as detected when the constant amount of M5B4 was allowed to compete with varying twofold dilutions of A11. There was no change in the optical density following the dilution of the A11 indicating that it did not compete for the same site on PV GP as shown in Figure 7. Antirabies diabody was used as a positive control [20].

### 3.8. Cross Reactivity of A11 to Unrelated Proteins

**Figure 8:**
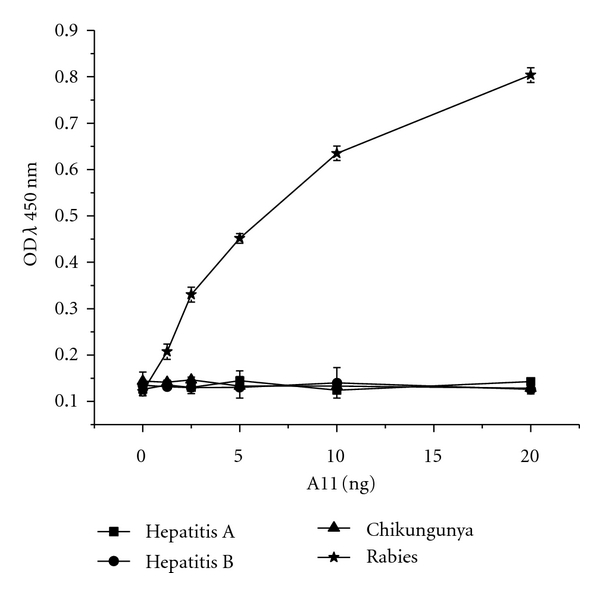
ELISA-based comparison of A11 (at different concentrations) binding to unrelated viruses showing that the antibody binds to rabies virus and does not show any reactivity to Hepatitis A virus (HAV), Hepatitis B surface antigen (HB), and Chikungunya virus (CHIKV). The data shown are the representative of three similar experiments and are the mean of triplicate samples.

Antigen specificity of the A11 was verified by reactivity in ELISA against hepatitis A, hepatitis B, and chikungunya viruses. The comparison of the absorbance values, as shown in Figure 8, indicated that A11 reacted only with rabies virus in a concentration-dependent manner and showed no reactivity towards other viruses.

### 3.9. Epitope Mapping

A constrained heptapeptide library displayed on filamentous phage was screened on coated scFv A11. Following four rounds of panning/selection, 10 clones were selected of which 7 bound to the same constrained heptapeptide (SGPSYTT). A sequence similarity between the constrained peptide recognized by A11 and the conformational antigen site II of PV GP at nucleic acid sequence 3489–3503 (SGFSY) strongly suggested that A11 bound to the antigenic site II of PV GP.

### 3.10. Amplification of Various Regions of the Glycoprotein Gene and Cloning into pGEX 4T1

PV GP was used as a template for the PCR amplification (94°C for 1 min, 50°C 1 min, 72°C 1 min, for 34 cycles) of the epitope regions (E1, E2, E3, E4 and E5) (Table 2(a)) by respective gene-specific primers (Table 2(b)) yielded 322 bp, 247 bp, 238 bp, 245 bp, and 316 bp amplicons, respectively (data not shown). The resultant epitope regions were digested with *Bam*HI and *Eco*RI and ligated into the bacterial expression vector pGEX 4T_1_ to yield pGEX 4T_1_ E1–E5.

### 3.11. Expression and Purification of Glycoprotein Epitope Regions

pGEX 4T_1_ E1–E5 containing glycoprotein epitopes (E1–E5) was transformed into BL21 (DE3) cells and was expressed by induction with 1 mM IPTG. The cell pellet was lysed, and the cytoplasmic fraction was purified on a glutathione agarose column. Analysis of the purified GST fusions E1–E5 by coomassie staining and immunoblotting (data not shown) indicated the presence of a 38, 35, 35, 35, and 37 kDa bands, respectively. The yield of glycoprotein epitope regions E1–E5 ranged from 1.8 to 2.0 mg/liter of culture.

### 3.12. Indirect ELISA for Epitope Specificity

**Figure 9:**
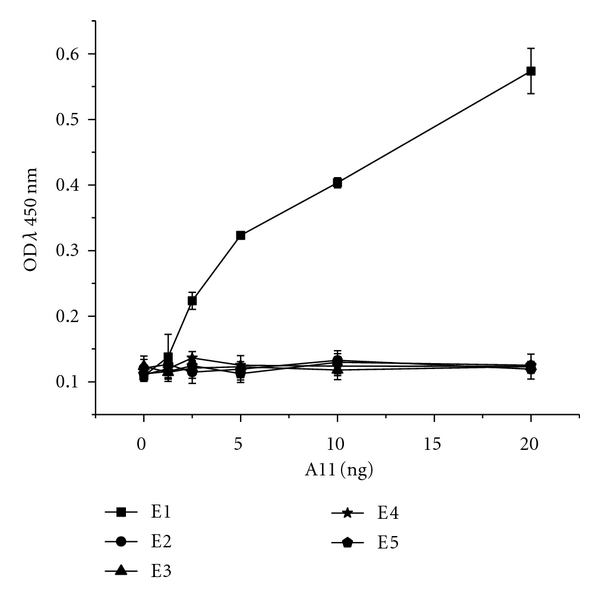
Analysis of epitope-binding affinity of A11 by ELISA to evaluate the activity of A11 to rabies virus glycoprotein epitopes. The data shown are the representative of three similar experiments and are the mean of triplicate samples.

Indirect ELISA was performed to determine the specificity of A11 towards E1–E5. Titration of the A11 against a constant dilution of different polypeptides of PV GP revealed a concentration-dependent reduction of the binding signal in the wells coated only with E1 as shown in Figure 9, indicating that A11 binds strongly with E1 epitope, which codes for the aminoacid sequence SGFSY present in the antigenic site II of PV GP.

## 4. Discussion

Antibody engineering using phage display technology [22] allowed the expression of scFvs on the surface of bacteriophage [12] thereby offering several advantages over hybridoma technology which included generation of antibodies with increased affinity and specificity by mimicking affinity maturation in normal immune system. This provided an antibody with a stable genetic source, which could be easily manipulated allowing their usage in various clinical diagnosis and therapeutic applications [23–28]. An enormous number of antibody fragments (upto 10^9^ clones) could be isolated from a single immunized animal, and the affinity of these antibodies could be increased by *in vitro* affinity maturation [29, 30].

In this study, we constructed an scFv library from murine splenocytes, which was directed against the PV GP using phage display technology. scFvs were expressed on the phage as membrane-anchored proteins, which allowed us to perform antibody selection very efficiently and further allowed the isolation of the best candidate through biopanning. Following three rounds of biopanning, 30 PV GP positive phage clones were selected by phage ELISA of which only 2 clones elicited a very strong signal in ELISA. DNA sequencing of these clones identified them as variable antibody genes belonging to the IgG1 subgroup and showed 100% similarity in their sequences. One was selected for further studies.

 The scFvs are expressed on the phage surface fused to the minor coat protein III, separated by an amber codon in TG1 strain of *Escherichia coli*, which facilitated numerous cycles of selection and infection. To achieve optimum expression of A11 as a soluble product, phagemid isolated from positive clones were amplified with primers containing restriction sites, and the resultant PCR product was cloned into a bacterial expression vector pET 20 b. Expression in *E. coli *resulted in the expression of a soluble ~28 kDa protein A11, which was purified to ~90% homogeneity using IMAC. Purification of the bacterial lysate resulted in the yield ~4 mg of purified A11/liter of culture with a homogeneity of ~90–95%. The ease of purification reiterated the fact that expression of a functional recombinant antibody in bacteria offered many advantages over the maintenance of a hybridoma cell line, which included minimal batch-to-batch variation, ease of scale up, and so forth, at a reasonable cost. The binding of A11 to PV GP in ELISA indicated proper folding of the protein following expression. A11 binding to PV GP was demonstrated by western blot analysis. Specificity study showed that the A11 reacted only with PV GP but not with other viruses as indicated by sandwich ELISA. A11 did not compete with M5B4 for binding to PV GP, suggesting that A11 and M5B4 bound to different epitopes. 

A large number of other researchers have mapped the various antigenic sites using mutational analysis on the glycoprotein of MAb-resistant variants [31–35], and they include antigenic site I located at aa 231 [36], antigenic site II, which is a discontinuous conformational epitope comprising aa 34 to 42 and aa 198 to 200 antigenic site III which is a continuous conformational epitope at aa 330 to 338 and is involved in viral pathogenicity [37, 38], and antigenic site IV is known to harbor overlapping linear epitopes [39–41]. The binding site of A11 was mapped using a constrained phage display library to facilitate the chance of selecting conformational peptides, which were complementary to the A11 paratope. The disulfide-constrained heptapeptides were expressed at the N-terminus of the pIII protein of the filamentous phage with the first cysteine preceded by an alanine residue, the second cysteine followed by short spacer (Gly-Gly-Gly-Ser), and the wildtype pIII sequence in tandem [42–44]. A11 bound to the C-SGPSYTT-C which showed sequence similarity with the PV GP site II (SGFSY) corresponding to the nucleic acid position 3489–3503 of the PV GP gene. Further, the binding of A11 on native PV GP was determined by expressing truncated fragments based on the known gene sequences of PV GP. Fragments of antigen gene were amplified and cloned into pGEX 4T_1_ bacterial expression vector and expressed as a GST fusions E1–E5. The fragments of the antigen were expressed and purified by affinity chromatography using glutathione-agarose, which permits rapid, mild, nondenaturing, and highly selective purification of binding enzymes such as GST. An ELISA study of these purified fragments clearly indicated that the selected A11 bound to E1, which encompassed the site II of PV GP and included the site SGFSY.

In conclusion, a mouse immune scFv library was constructed from splenocytes of PV immunized mice. The constructed mouse scFv library revealed and confirmed the broad diversity by *Bst*NI fingerprinting and DNA sequence analysis. The selected scFv fragment A11 was shown to bind to site II of PV GP both by the use of a constrained library and a truncated PV GP encompassing the site II. The data presented not only defined the functional aspects of the scFv but also reiterated the fact that this methodology could be used as a means for quick development of recombinant antibodies against rabies and other infectious diseases. The antibodies thus developed could be used not only for postexposure prophylaxis of rabies but would also allow us to address the issue of natural variation amongst rabies virus field isolates.

## Figures and Tables

**Table 1:** cmd Primers used for PCR of VH and VL regions and SOE PCR for construction of scFv.

Variable light chain forward primers	
LB1	GCCATGGCGGA(CT)ATCCAGCTGACTCAGCC
LB2	GCCATGGCGGA(CT)ATTGTTCTC(AT)CCCAGTC
LB3	GCCATGGCGGA(CT)ATTGTG(AC)T(AC)ACTCAGTC
LB4	GCCATGGCGGA(CT)ATTGTG(CT)T(AG)ACACAGTC
LB5	GCCATGGCGGA(CT)ATTGT(AG)ATGAC(AC)CAGTC
LB6	GCCATGGCGGA(CT)ATT(AC)AGAT(AG)A(AC)CCAGTC
LB7	GCCATGGCGGA(CT)ATTCAGATGA(CT)(AGT)CAGTC
LB8	GCCATGGCGGA(CT)AT(CT)CAGATGACACAGAC
LB9	GCCATGGCGGA(CT)ATTGTTCTCA(AT)CCAGTC
LB10	GCCATGGCGGA(CT)ATTG(AT)GCT(GC)ACCCAATC
LB11	GCCATGGCGGA(CT)ATT(GC)T(AG)ATGACCCA(AG)TC
LB12	GCCATGGCGGA(CT)(AG)TT(GT)TGATGACCCA(AG)AC
LB13	GCCATGGCGGA(CT)ATTGTGATGAC(GCT)CAG(GT)C
LB14	GCCATGGCGGA(CT)ATTGTGATAAC(CT)CAGGA
LB15	GCCATGGCGGA(CT)ATTGTGATGACCCAG(AT)T
LB16	GCCATGGCGGA(CT)ATTGTGATGACACAACC
LB17	GCCATGGCGGA(CT)ATTTTGCTGACTCAGTC

Variable light chain reverse primers	

LF1	GGAGCCGCCGCCGCCAGAACCACCACCACCAGAACCACCACCACCACGTTTGATTTCCAGCTTGG
LF2	GGAGCCGCCGCCGCCAGAACCACCACCACCAGAACCACCACCACCACGTTTTATTTCCAGCTTGG
LF4	GGAGCCGCCGCCGCCAGAACCACCACCACCAGAACCACCACCACCACGTTTTATTTCCAACTTTG
LF5	GGAGCCGCCGCCGCCAGAACCACCACCACCAGAACCACCACCACCACGTTTCAGCTCCAGCTTGG

Variable heavy chain forward primers	

HB1	GGCGGCGGCGGCTCCGGTGGTGGTGA(GT)GT(AG)(AC)AGCTTCAGGAGTC
HB2	GGCGGCGGCGGCTCCGGTGGTGGTGAGGT(GCT)CAGCT(GCT)CAGCAGTC
HB3	GGCGGCGGCGGCTCCGGTGGTGGTCAGGTGCAGCTGAAG(GC)A(GC)TC
HB4	GGCGGCGGCGGCTCCGGTGGTGGTGAGGTCCA(AG)CTGCAACA(AG)TC
HB5	GGCGGCGGCGGCTCCGGTGGTGGTCAGGT(CT)CAGCT(GCT)CAGCA(AG)TC
HB6	GGCGGCGGCGGCTCCGGTGGTGGTCAGGT(CT)CA(AG)CTGCAGCAGTC
HB7	GGCGGCGGCGGCTCCGGTGGTGGTCAGGTCCACGTGAAGCAGTC
HB8	GGCGGCGGCGGCTCCGGTGGTGGTGAGGTGAA(GC)(GC)TGGTGGAATC
HB9	GGCGGCGGCGGCTCCGGTGGTGGTGA(AGC)GTGA(AT)G(CT)TGGTGGAGTC
HB10	GGCGGCGGCGGCTCCGGTGGTGGTGAGGTGCAG(GC)(GT)GGTGGAGTC
HB11	GGCGGCGGCGGCTCCGGTGGTGGTGA(GT)GTGCA(AC)CTGGTGGAGTC
HB12	GGCGGCGGCGGCTCCGGTGGTGGTGAGGTGAAGCTGATGGA(AG)TC
HB13	GGCGGCGGCGGCTCCGGTGGTGGTGAGGTGCA(AG)CTTGTTGAGTC
HB14	GGCGGCGGCGGCTCCGGTGGTGGTGA(AG)GT(AG)AAGCTTCTCGAGTC
HB15	GGCGGCGGCGGCTCCGGTGGTGGTGAAGTGAA(AG)(GC)TTGAGGAGTC
HB16	GGCGGCGGCGGCTCCGGTGGTGGTCAGGTTACTCT(AG)AAAG(AT)GT(GC)TG
HB17	GGCGGCGGCGGCTCCGGTGGTGGTCAGGTCCAACT(AGC)CAGCA(AG)CC
HB18	GGCGGCGGCGGCTCCGGTGGTGGTGATGTGAACTTGGAAGTGTC
HB19	GGCGGCGGCGGCTCCGGTGGTGGTGAGGTGAAGGTCATCGAGTC

Variable heavy chain reverse primers	

HF1	ATGCGCGGCCGCCGAGGAAACGGTGACCGTGGT
HF2	ATGCGCGGCCGCCGAGGAGACTGTGAGAGTGGT
HF3	ATGCGCGGCCGCCGCAGAGACAGTGACCAGAGT
HF4	ATGCGCGGCCGCCGAGGAGACGGTGACTGAGGT

**Table:** (a)

Epitope gene ID	Location on PV GP	Size in bp
E1	3318–3640	322
E2	3641–3888	247
E3	3889–4134	238
E4	4135–4380	245
E5	4381–4697	316

**Table:** (b)

Epitope 1 For	ATCGGGATCCGGAGTATTTTTCAATGGTATAAT
Epitope 1 Rev	ATCGGAATTCTCCTCTGAGATTGTGTTGT
Epitope 2 For	ATCGGGATCCGGAGTATTTTTCAATGGTATAAT
Epitope 2 Rev	ATCGGAATTCCCGTTCGTGCACATCG
Epitope 3 For	ATCGGGATCCAAGAACGGTGACGAGG
Epitope 3 Rev	ATCGGAATTCCCGTTCGTGCACATCG
Epitope 4 For	ATCGGGATCCCAGCAACATATGGAGTTGT
Epitope 4 Rev	ATCGGAATTCCAAGGCAGTCAGGGCC
Epitope 5 For	ATCGGGATCCTGCCCAAACAATTTGGTA
Epitope 5 Rev	ATCGGAATTCCCCGTTCATTTTTATGGC

## Abbreviations

cDNA: Complementary DNA
DAB: 3, 3′ diaminobenzidine
DNA: Deoxyribonucleic acid
GP: Glycoprotein
OD: Optical density
RNA: Ribonucleic acid
RT-PCR: Reverse transcription-polymerase chain reaction
scFv: single-chain Fv antibody fragment
VH: Variable heavy chain
VL: Variable light chain.
